# Wernicke’s encephalopathy in patient with alcohol dependence

**DOI:** 10.1192/j.eurpsy.2025.927

**Published:** 2025-08-26

**Authors:** M. P. Pando Fernández, P. Martínez Gimeno, M. Calvo Valcárcel, M. A. Andreo Vidal, M. D. L. Á. Guillén Soto, M. B. Arribas-Simón, C. Alario Ruiz, O. M. Segurado Martín, G. Guerra Valera, M. Fernández Lozano, M. J. Mateos Sexmero, N. Navarro Barriga, B. Rodríguez Rodríguez, A. Aparicio Parra, G. Lorenzo Chapatte, M. Rios Vaquero, L. Rojas Vázquez, A. Monllor Lazarraga, L. Sobrino Conde, F. J. González Zapatero, L. del Canto Martínez, M. E. Espinosa Muth

**Affiliations:** 1 Psiquiatría; 2 HCUV, Valladolid, Spain

## Abstract

**Introduction:**

The worldwide prevalence of Wernicke-Korsakoff syndrome is thought to range from 0-2%. Those at greatest risk include the homeless, the elderly, and psychiatric patients (1). In treatment, typical regimens include high doses of intravenous thiamine, three times daily for at least three days. Electrolyte abnormalities should be corrected and fluids replaced.

**Objectives:**

We are interested in studying the evolution of a patient with alcohol withdrawal syndrome progressing to wernicke’s encephalopathy.

**Methods:**

We conducted a literature review by searching for articles in Pubmed.

**Results:**

A 40-year-old male, with no medical or surgical history of interest, alcohol consumer, was admitted to the hospital ICU for an episode of ataxia and agitation in the context of four days of alcohol abstinence. He was sedated and orotracheal intubation was performed and treatment was started with thiamine, tiapride and diazepam. After hemodynamic and respiratory stability, the patient was transferred to the Internal Medicine ward where he presented clinical symptoms compatible with Wernicke’s Encephalopathy (cerebellar ataxia and nystagmus). Psychiatry was consulted to adjust treatment and to carry out a psychosocial approach for discharge (alcohol withdrawal center).

The patient’s evolution has been favorable with the adjustment of psychopharmacological treatment. In the neurological examination we observed nystagmus and cerebellar ataxia. In the psychopathological examination the suspicious contact, psychomotor restlessness, mild generalized tremor in both MMSS are remarkable. Speech difficult to understand due to language barrier. Traits of impulsivity in the foreground. Unstructured biological rhythms. Partial insight. Intellectual functions and volitional abilities preserved.

In the complementary tests without significant remarkable alterations. In the treatment adjustment, a de-escalation of diazepam has been carried out for discharge. Treatment with pregabalin, tiaprizal, thiamine and vitamins B1-B6-B9 was also prescribed. Recommendation of absolute cessation of alcohol consumption and follow-up by internal medicine, psychiatry and social work.

**Conclusions:**

Wernicke-Korsakoff syndrome is a clinical diagnosis and Wernicke’s encephalopathy should be suspected in any person at risk of thiamine deficiency presenting oculomotor findings, ataxia or confusion (1). Thus, in our patient presenting ataxia and nystagmus in the context of alcohol abstinence and some malnutrition, an early approach with thiamine can be performed to prevent progression to Korsakoff’s syndrome.

Once amnesia and executive deficit are present, Korsakoff’s syndrome should be suspected. The key to good outcomes is therefore to detect Wernicke’s encephalopathy early and treat it with thiamine (1). Severe concomitant infections, including sepsis of unknown origin, are frequent during Wernicke’s phase (2). In our patient there were no complications.

**Disclosure of Interest:**

None Declared

